# Ten simple rules for fostering creativity in research labs

**DOI:** 10.1371/journal.pcbi.1012788

**Published:** 2025-02-14

**Authors:** Matthias C. Rillig

**Affiliations:** 1 Freie Universität Berlin, Institute of Biology, Berlin, Germany; 2 Berlin-Brandenburg Institute of Advanced Biodiversity Research, Berlin, Germany; Dassault Systemes BIOVIA, UNITED STATES OF AMERICA

## Abstract

Research lab groups are hotspots for the education of the next generation of scientists, and making these units work as creatively as possible is essential for solving pressing issues in biology, the environment, and beyond. This article highlights 10 points that can help make labs as creative as possible. Several of these points are about setting up a creative lab culture; others are about fostering group-level creative output, some are more about encouraging creativity of individual team members, or both. While the head of a research group, the principal investigator, plays an important role, this can only be successful in healthy labs where everyone contributes.

## Introduction

We may be in a period of declining disruptiveness of research [1], and while many reasons likely contribute to this phenomenon, this observation serves as a reminder to keep championing the role of creativity in research [2], especially in educating the next generation of researchers. Research labs or teams, by which I mean individual groups headed by a principal investigator (PI), are key for creativity to unfold in the research process [3,4], and they are also the hotspot of graduate student and postdoctoral training. Labs provide the setting for group-level creativity [5,6], and this setting also either encourages (or not) the individual creativity of team members. I use creativity in the usual way here, namely, as the connection of known components that leads to something novel and useful within a given domain [7]. Creativity can enter into the scientific process at various points [8], and the 10 rules that follow apply to all of these: during inception of ideas for studies, during experimental design, data analysis, data presentation, for the dissemination of results to a broader audience [9] and potentially for the potential application of results. This point is important to keep in mind, since it is not just about coming up with ideas for research, which is what most people will generally associate with creativity in the scientific context, at least in my experience. Also, creativity can very much be enhanced and trained [2,10], it is not just a given trait of certain people, and it is responsive to the general setting. So, how might we enhance creativity within a lab group?

**Fig 1:**
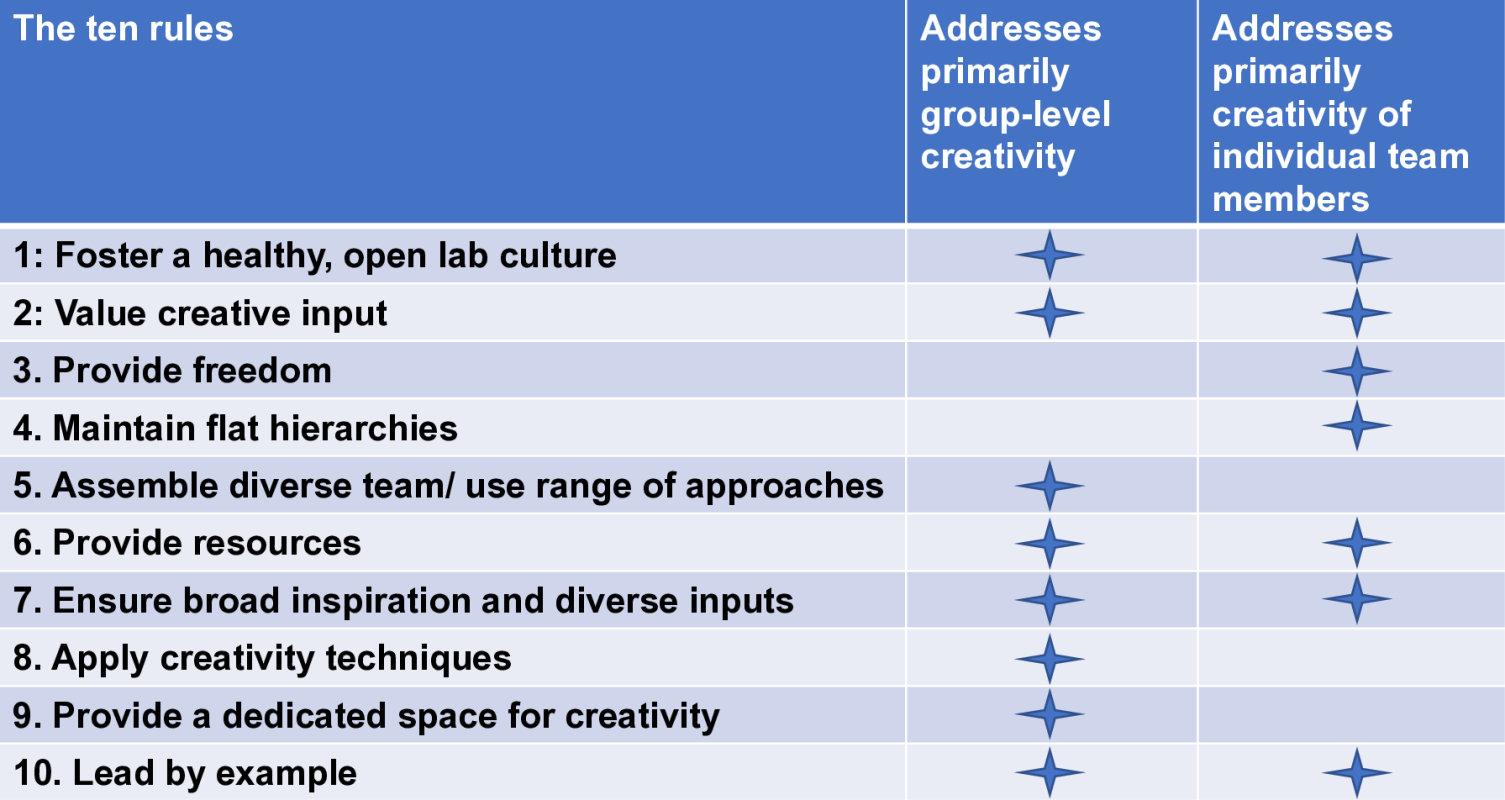
Ten simple rules for fostering creativity in research labs. For each of the 10 rules, it is indicated if they primarily address group-level or individual team member creative output.

For context, the author is the head of an ecology research lab (about 50 people) at a university, and not a creativity researcher. Creativity research, at the individual and team level, is a rapidly expanding field spanning several disciplines including psychology [11], neuroscience [12], and organizational research [13]. Here, the article is intended as a starting point for discussion in lab groups and aimed at early career researchers in the sciences, as well as PIs in the biology field. Conditions will differ among different settings, institutional contexts and also different fields, and thus may require tailored approaches. With this in mind, here are 10 simple rules (Fig 1), some are aimed more at group-level creativity or creativity of individual team members, or both. It is impossible to force creativity, one needs to allow it to unfold by offering the right setting [14,15]. Following these rules will help with this.

## Rule 1: Foster a healthy, open lab culture

Providing a healthy lab is the most important rule, since the right climate and environment creates the basic setting for everything else. The lab should be a place where everyone feels safe and comfortable with sharing their ideas, it should be a healthy lab environment [16]. This is important in lab meetings or seminars, but also in day-to-day operations. The general “social climate” of a team or organization is important for creativity [17]. In teams with a problematic climate (sometimes also referred to as “toxic” labs or workplaces [18]), people will still have ideas, but they will often not come to light. If labs are not healthy workplaces, people’s minds are likely busy with other things, like how to overcome the situation, and thus creativity overall tends to be stifled [19]. Key aspects of a healthy lab are a focus on the well-being of lab members, an emphasis on team building and trust, and on promoting the professional development of team members; healthy labs encourage collaboration and respectful and friendly communication. This is the environment where people will be willing to share their own ideas and where they are receptive to the ideas of others.

## Rule 2: Value creative input

It helps when people have a feeling that their creative input is openly received, valued and rewarded. This appreciation should come from the PI, but also from other group members, the peers. A sure way to have good ideas is to have many ideas from which to pick, and thus the effort towards developing output is what should be appreciated, not just the brilliant idea that may arise. Doing this is pretty simple: thank people that actively contribute to the discussion, and let them know that you value their creativity; let people know that you appreciate their figures in manuscripts, when they are little masterpieces; reward them by including them as co-authors on papers you develop (this requires taking notes during discussions to keep track of contributions). All this will send the message that creativity in this lab is valued. No, ideas are not “a dime a dozen”, as people sometimes say; and certainly not good ideas: it is important to properly value them.

## Rule 3: Provide freedom

Most lab members will have assigned tasks; for example, their primary responsibility is to fulfill the milestones of a grant or fellowship. But beyond that, when new members join my lab, they are told that when goals have been achieved or are well under way, they have the freedom to roam, explore other topics, and pursue their interests. Communicating this clearly and frequently is very important. As individual lab members are encouraged to pursue what they are curious about and are given the freedom to do so, they can develop their most creative output. This idea exemplified in the corporate world by Google’s 80/20 rule, which allows employees to spend 20% of their time on their own projects and 80% on assigned tasks. This rule has the goal of spurring creativity. There is a certain risk that such side projects take over, so it is important to keep communicating and to make sure the primary responsibilities are met.

## Rule 4: Maintain flat hierarchies

Strong hierarchical settings can stifle creative input by team members, because people may be hesitant to speak up. Hierarchies can exist not just from PI to the lab team, but also within the lab team (for example, between senior postdocs and PhD or MSc students). My PhD advisor once told me: I expect you to show me that I’m wrong. I only later realized how powerful that was. Strong hierarchies in a lab can be unhelpful in many ways, but they certainly do not help with creative idea development and especially communication of these ideas. Nevertheless, it should not be overlooked that a certain hierarchical organization is helpful in defining goals and clarifying roles and responsibilities within the team [20].

## Rule 5: Assemble a diverse team using a range of approaches

Creativity comes from connecting dissimilar points in new and useful ways and one way to reach this diversity of input and processing is by working with a diverse group of people [21]. This diversity can manifest in any number of dimensions, including country of origin, culture, gender, life experience, and diversity of scientific background. This diversity along any number of axes can lead to everyday encounters in the lab that lead to unusual connections and ideas. The diversity in people, especially in terms of different scientific backgrounds, can also lead to the lab pursuing a range of different approaches and research questions within the lab’s general scope, like theoretical, modeling, experimental, observational approaches; some projects will be more applied, some will be more foundational in nature; and perhaps they will use different model systems or settings. This means that there can be a lot of exchange within a lab (especially larger lab groups, or joint labs) because of this diversity of questions within a common overall mission, leading to new connections. There are other advantages to labs using a range of different research approaches, not the least of which is increased resilience in times of crises [22]; but the biggest advantage is the potential for making unusual links just because people sit together during lunch breaks. However, it is important to highlight that diversity also can lead to conflict in teams [23,24]; and thus increasing diversity does not necessarily lead to increased creativity, if the team is not properly managed.

## Rule 6: Provide resources

To a certain degree, lab financial resources can be important for creative output. If a lab has sufficient funding to explore new ideas, then this funding can lead to more ideas being realized. With this sort of discretionary funding, lab members can, for example, conduct first experiments tinkering with their ideas, even if they are just simple, exploratory studies. When such experiments yield promising results, this success can in turn fuel more ideas that build on the outcome from these pioneering studies. Having funding available also means having the resources to extend contracts for people to explore such ideas once a promising new line of inquiry has opened up. Financial resources can also be important to allow team members to receive specific training elsewhere, for example, to visit another lab or to attend a workshop, if the developing idea demands it. Therefore, it is important to secure lab financial reserves, if possible, for example, from indirect cost refunds or other sources that allow more freedom in spending; and to use this money explicitly for this purpose.

## Rule 7: Ensure broad inspiration and diverse inputs

Having a broad range of input is crucial, since this can provide the starting points to be connected in a creative act [15]. This diverse input can be achieved by reading papers (like in a journal club or discussion group) that are outside of the specific research field of the lab group, or by encouraging (or hiring) people with broad interests. A further option is hosting interdisciplinary workshops or presentations, or attending talks in other departments or at conferences. One way to leverage conference experiences is to ask lab members who attended a meeting to report briefly to the lab group what they found most exciting. Another effective way of achieving a diverse input is to work with visitors, for example, artists, such as in artists-in-residence programs [25]. If your department or university does not have one of these, perhaps you can start one. Several research ideas have come from such interactions with artists in our group [26]. In our lab group, we also have recently welcomed our first philosopher-in-residence. Also, sabbatical visitors can add a lot of new perspectives, especially if they work on somewhat different topics or approaches. For example, our ecology lab has enormously profited from a longer-term visit by a polymer chemist, in ways that were impossible to predict. The key is to allow for a visit to be long enough to provide opportunities for meaningful exchanges, and this is typically several weeks to months. Broad buy-in by the group for interacting with such visitors is also vital, it is the difference between success and failure.

## Rule 8: Apply creativity techniques

There is a whole arsenal of creativity techniques [7], yet we mostly seem to be stuck with brainstorming, for example, in our lab meetings. But have you heard of brainwriting (a written version of brainstorming), the Six Thinking Hats [27], the Zwicky box (a.k.a. morphological analysis) [28], or the SCAMPER technique [29]? There are many creativity-enhancing techniques, both for individual-level creativity and for group settings. These techniques can be used during lab meeting discussions or workshops. There are also a lot of online resources out there, and a whole industry caters to the need for this material, mostly geared towards a corporate audience, but with minor adjustments they are also well-suited to a research lab. It can be fun to try these out and test what works for your lab.

## Rule 9: Provide a dedicated space for creativity

It is extremely helpful if there is a specific space for expressing creative ideas [30]. Such a space can be provided during lab meeting; for example, at the end of discussing a paper in our journal club, we always dedicate the last 15–30 min to discussing ideas related to what we just read. What ideas has this paper given us, how could we build on this research or apply it to our situation? We typically also discuss creative aspects within the work we read. For example, what aspect of the paper did we find most creative and why? What can we learn from this aspect? Can a particular example of creativity within the paper be generalized? Or there can be specific workshops or dedicated idea-generating sessions, either based on literature we have read or other forms of input. Perhaps this space can take the form of a physical or online drop-box, where notes containing ideas can be deposited, if needed also anonymously. The most important thing is perhaps the conditioning trigger for the brain: at a certain point of a meeting or on other occasions we focus on creativity, and it is part of a routine, so everybody knows it’s coming.

## Rule 10: Lead by example

Leadership can be important for team creativity, empowering others to achieving creative outcomes, given a certain context [31–33]. PI or senior group members can lead by example, showing that they strive to develop unusual ideas; this can be achieved by sharing their thinking, by engaging in creative activities, or by taking an interest in how creative ideas arise. I also teach a course with lectures and exercises on creativity in research (in the environmental sciences) to MSc students. Through teaching this course, I am learning more about this topic, and this also signals that I as the PI am interested in creativity. Whatever you do as a PI, or as a postdoc or graduate student in the lab, it will help if you show interest in creative approaches, if you are open to learning about creativity and how it can be enhanced. I think the message will be received by the team.

## Conclusions

Don’t be fooled by the “simple” in the title, pre-determined by the theme of this article collection, as following some of these suggestions is anything but straightforward. Many of the rules are about creating a certain lab culture (rules 1–4), and this does not happen overnight. It is also important to recognize that even though the PI plays an important role (see, for example, rule 10), also as the person who sets the stage for the kind of lab into which a group of people develops, none of this works without the cooperation of the lab members [34]. Making labs more creative takes everybody.
